# Molecular Epidemiology of SARS-CoV-2 in Northern Greece from the Index Case up to Early 2025 Using Nanopore Sequencing

**DOI:** 10.3390/epidemiologia6040078

**Published:** 2025-11-12

**Authors:** Georgios Meletis, Styliani Pappa, Georgia Gioula, Maria Exindari, Maria Christoforidi, Anna Papa

**Affiliations:** Laboratory of Microbiology, School of Medicine, Aristotle University of Thessaloniki, 54124 Thessaloniki, Greece; s_pappa@hotmail.com (S.P.); ggioula@auth.gr (G.G.); mexidari@auth.gr (M.E.); maria.christ94@gmail.com (M.C.); annap.med@gmail.com (A.P.)

**Keywords:** SARS-CoV-2, COVID-19, molecular epidemiology, genomic surveillance, VOC, alpha, beta, delta, omicron, EG.5, JN.1, KP.3, recombination, nanopore, Greece

## Abstract

Background/Objectives: Since its emergence in late 2019, SARS-CoV-2 has demonstrated remarkable genetic diversity driven by mutations and recombination events that shaped the course of the COVID-19 pandemic. Continuous genomic monitoring is essential to track viral evolution, assess the spread of variants of concern (VOCs), and inform public health strategies. The present study aimed to characterize the molecular epidemiology of SARS-CoV-2 in northern Greece from the first national case in February 2020 through early 2025. Methods: A total of 66 respiratory samples collected from hospitalized patients across Northern Greece were subjected to whole-genome sequencing using Oxford Nanopore Technologies’ MinION Mk1C platform and the ARTIC protocol. Sequences were analyzed with PANGO, Nextclade, and GISAID nomenclature systems for lineage and clade assignment, and the WHO nomenclature for VOCs. Results: Across 66 genomes, 34 PANGO lineages were identified. Early introductions included B.1 (2/66), B.1.177 (3/66), and B.1.258 (1/66). Alpha (5/66) and Beta (5/66) circulated in February–June 2021. Delta (AY.43) was detected in early 2022 (2/66; Jan–Feb) but was rapidly displaced by Omicron and reached 100% of the sequences by May 2022. Omicron diversified into BA.1/BA.1.1 (3/66), BA.2 (6/66), BA.4/BA.5 (14/66), BF.5 (1/66), EG.5 (1/66; designated a WHO Variant of Interest in 2023), JN.1 (4/66; globally dominant lineage prompting vaccine updates in 2024–2025), KS.1 (2/66; together with KS.1.1 are recognized PANGO lineages that were tracked internationally but remained less prevalent), KP.3 (5/66; together with KP.3.1.1, prominent “FLiRT” descendants circulating in 2024), and recombinants XDK, XDD, and XEC (5/66), reported by their PANGO names in accordance with the WHO’s current framework, which reserves Greek letters only for newly designated VOCs. Conclusions: This five-year genomic analysis provides an insight into the continuous evolution of SARS-CoV-2 in northern Greece. The findings underscore the importance of sustained genomic surveillance, integrated with epidemiological data, to detect emerging variants, monitor recombination, and strengthen preparedness for future coronavirus threats.

## 1. Introduction

Severe acute respiratory syndrome coronavirus 2 (SARS-CoV-2) has spread globally after emerging in China in 2019 and caused the coronavirus disease (COVID-19) pandemic with millions of deaths and enormous social and economic implications [1]. Since then, COVID-19 presented with variable symptomatology and severity (ranging from asymptomatic to acute respiratory distress syndrome) depending on the circulating variants and the interaction of the virus with human hosts [2].

SARS-CoV-2 is a single-stranded RNA virus of the genus Betacoronavirus in the family *Coronaviridae*. Over time, it has been subjected to continuous point-mutations that consequently resulted in the emergence of different lineages and variants [3]. Thus, during the pandemic and afterwards, numerous variants with different mutation combinations and variable clinical characteristics have emerged [4], posing the need for monitoring, classification and nomenclature systems.

The Global Initiative on Sharing All Influenza Data (GISAID) was established in 2008, in response to the H5N1 influenza pandemic, and its initial aim was to provide open access to genomic data of influenza viruses [5]. Following the identification of COVID-19 as a newly emerging viral respiratory disease, GISAID established the EpiCoV™ platform, to ensure open access to data and to overcome hurdles and restrictions which discouraged or prevented prompt dissemination of virological data prior to official publication. Nowadays, GISAID maintains the world’s largest repository of SARS-CoV-2 sequences, including related clinical, epidemiological and geographical data.

The Nextstrain pathogen surveillance platform (Nextstrain.org) offers real-time views of evolving pathogen populations through interactive visualizations, enabling users to explore datasets and analyses that are continuously updated as new genomic data become available [6]. In early 2020, Nextstrain introduced informal clade labels for SARS-CoV-2 to facilitate URL links that provided an “automatic zoom” to specific regions of the phylogenetic tree. These labels, composed of adhoc letter–number combinations, were not intended to serve as a permanent naming system.

For SARS-CoV-2, the Phylogenetic Assignment of Named Global Outbreak (PANGO) dynamic nomenclature [7] is widely recognized as the standard system for classifying and naming genetically distinct lineages, based on analyses of complete or near-complete viral genomes. Within this framework, new lineages are assessed by a committee according to criteria such as novel evolutionary traits, transmissibility, pathogenicity, significant increases in frequency, and evidence of spread across regions [8]. Although scientifically rigorous, it was quickly acknowledged that the PANGO naming scheme could cause confusion, particularly in non-technical discussions or when conveying information to the general public.

The World Health Organization (WHO) established a classification system for SARS-CoV-2 variants based on their actual or anticipated clinical and epidemiological impact on public health (https://www.who.int/activities/tracking-SARS-CoV-2-variants accessed on 3 September 2025). Variants were grouped into categories such as variants under monitoring (VUM), variants of interest (VOI)—defined by features requiring ongoing surveillance and further assessment—and variants of concern (VOC), which demonstrated increased transmissibility, virulence, or pathogenicity, and in some cases showed partial resistance to treatment or immune protection from vaccination or prior infection [9,10,11]. To facilitate communication with healthcare providers and the general public, WHO also introduced a simplified nomenclature using Greek alphabet labels, applied only to major SARS-CoV-2 lineages that significantly influenced the course of the pandemic. The most clinically important variants designated with Greek letters were Alpha, Beta, Gamma, Delta, and Omicron. Since March 2023, WHO has emphasized the VUM, VOI and VOC categories and Greek letters were reserved only for newly designated VOCs. Later Omicron sublineages (e.g., EG.5, JN.1, KP.3, KS.1) are therefore reported by their PANGO names with their WHO category at the time of circulation and are not assigned new Greek letters.

The emergence of Omicron and its subsequent variants was associated with overall less virulence and triggered the end of the pandemic status. However, the molecular characterization of SARS-CoV-2was kept on by reference laboratories in order to monitor the evolution of the virus. Moreover, in March 2024, WHO launched a coronavirus network (CoViNet) to enhance coordination and support for the early, accurate detection, monitoring, and assessment of SARS-CoV-2, MERS-CoV, and emerging coronaviruses of public health significance (https://www.who.int/news/item/27-03-2024-who-launches-covinet--a-global-network-for-coronaviruses accessed on 3 September 2025).

The aim of the present study was to apply a next-generation sequencing (NGS) workflow for identifying various SARS-CoV-2 variants circulating in northern Greece, in order to obtain whole-genome sequences (WGS) from SARS-CoV-2-positive samples in various time periods starting from the first COVID-19 case in Greece and expanding up to early 2025.

## 2. Materials and Methods

### 2.1. Sample Collection and Preparation

The study was carried out at the Microbiology laboratory, School of Medicine, Aristotle University of Thessaloniki, Greece. Nasopharyngeal and oropharyngeal swabs were sent to the National Influenza Centre for Northern Greece from infected patients with SARS-CoV-2 hospitalized in various hospitals across northern Greece. Viral RNA was extracted using the Nucleic Acid Extraction Kit (Magnetic Bead Method) by Zybio Inc. (Chongqing, China) according to the manufacturer’s protocol, and a reverse transcriptase-polymerase chain reaction (RT-PCR) was applied for the detection of SARS-CoV-2 (TaqPath COVID-19 CE-IVD RT-PCR Kit, Thermo Fisher Scientific, Waltham, MA, USA). A total of 66 respiratory samples with Ct values of 20–29 in diagnostic RT-PCR were selected following a convenience strategy for NGS. All positive SARS-CoV-2 samples were stored at −80 °C until further use.

### 2.2. Library Preparation/Sequencing Using MinION Mk1C and GridION

An amplicon-based NGS protocol was applied to amplify overlapping segments of the viral genome of ~400 bp in length each, to obtain the complete genome sequence of SARS-CoV-2 (~30,000 bp). The sequencing was performed on the MinION platform of Oxford Nanopore Technologies (ONT, Oxford, UK). Various bioinformatic programs were applied, and the obtained sequences were compared with respective ones from the GISAID Bank.

In the present study we applied the PCR tiling of SARS-CoV-2 virus-classic (SQK-LSK109 with EXP-NBD104-114) sequencing protocol (Oxford Nanopore Technology-ONT, Oxford, UK) using the primers V3 and V4.1 developed by the ARTIC Network (https://artic.network accessed on 3 September 2025) and the third-party reagents from New England Biolabs (NEB, Ipswich, MA, USA). The consensus sequence was determined using reference-based alignment. All sequencing runs contained a negative control sample and were performed using the ligation sequencing kit 109 with Native Barcoding Expansion 1-12(EXP-NBD104) and 13-24(EXP-NBD114) (SQK-LSK109, EXP-NBD104-114, ONT, Oxford, UK). Before cDNA synthesis, the samples with Ct values 12–15 were diluted 1:100 with nuclease-free water; for Ct value15–18 they were diluted at 1:10, while when the Ct value was 18–32 they were used undiluted. cDNA synthesis was performed using 16 μL of viral RNA mixed with 4 μL of LunaScript RT SuperMix (M3010, NEB, Ipswich, MA, USA), following the manufacturers guidelines. Following cDNA synthesis, the overlapping amplicons (400 bp) were generated using primer pool V3 or V4.1 (ARTIC nCoV-2019 V3 Panel and ARTIC nCoV-2019 V4.1 Panel, IDT, Coralville, IA, USA). 15 ng of the prepared library was loaded in a total volume of 75 μL onto a primed R9.4.1 or R10.4 flow cell (ONT, Oxford, UK) installed in a MinION Mk1C or GridION (ONT, Oxford, UK) device and run for 18 h following the protocol of use as defined by the manufacturer.

Basecalling and demultiplexing were conducted using the MinKnow software 24.06.5 which is embedded within the MinION Mk1C and GridION devices (ONT, Oxford, UK), barcodes were trimmed. A first quick analysis was performed with the EPI2MEAgent cloud-based platform. Subsequent data analysis was also performed on the EPI2ME platform, developed by Metrichor Ltd. (Oxford, UK), incorporating Artic, Nextclade and Pagolin tools. The ARTIC pipeline assessed the depth of coverage for each barcoded sample and facilitates the examination of specific amplicons that may not have been successfully amplified by both primer pools. Nextclade identified genetic variants by comparison with the reference genome and provided quality control metrics. Pangolin was used to determine the lineage of each sample. The reference genome utilized for SARS-CoV-2 analysis was derived from the Wuhan strain (GenBank Accession number MN908947).

## 3. Results

Whole genome sequences (WGSs) were taken from sixty-six SARS-CoV-2 positive samples starting from February 2020, including the first COVID-19 case in Greece (Sample 141). Genome coverage greater than 90% and more than 30X depth were used as criteria for successful sequencing. All samples were classified according to PANGO, WHO (where applicable), GISAID and Nextstrain (Table 1).

**Figure 1:**
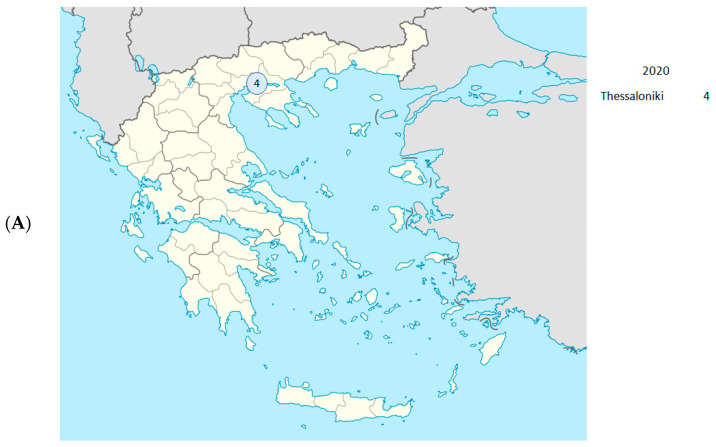

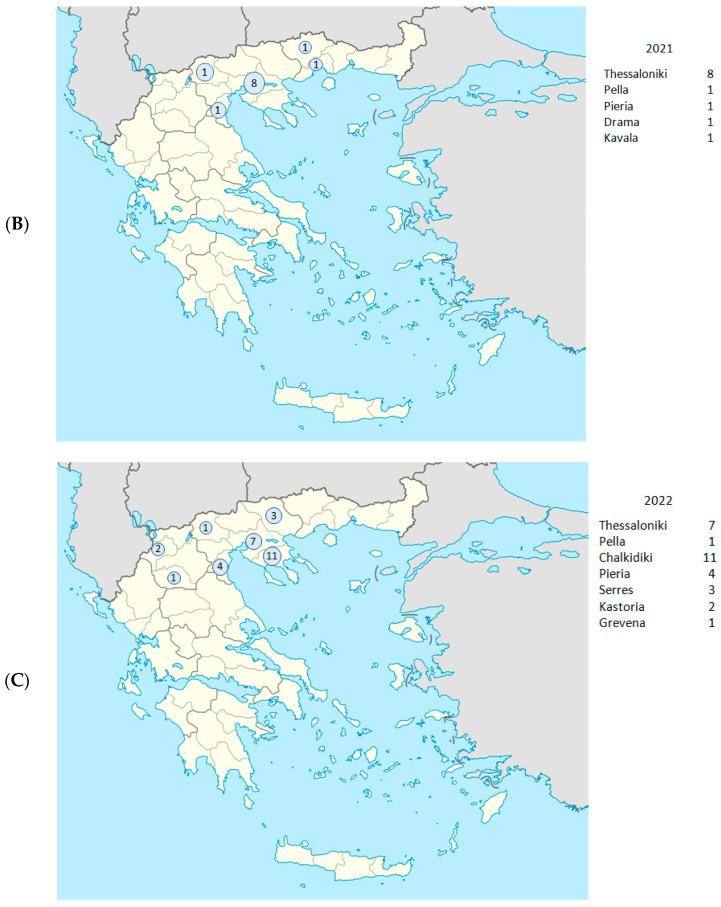

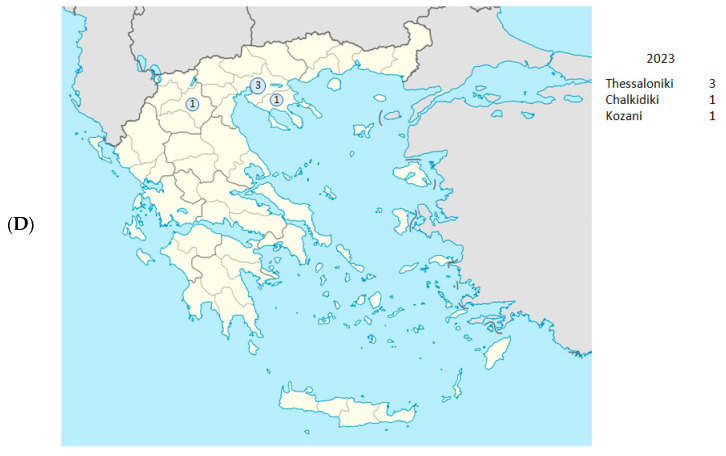

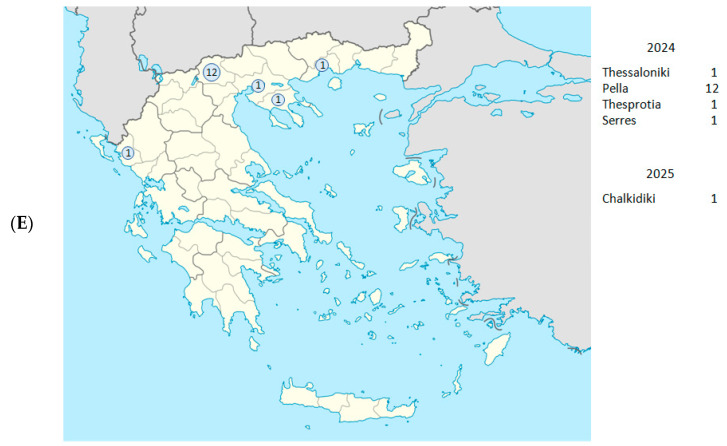

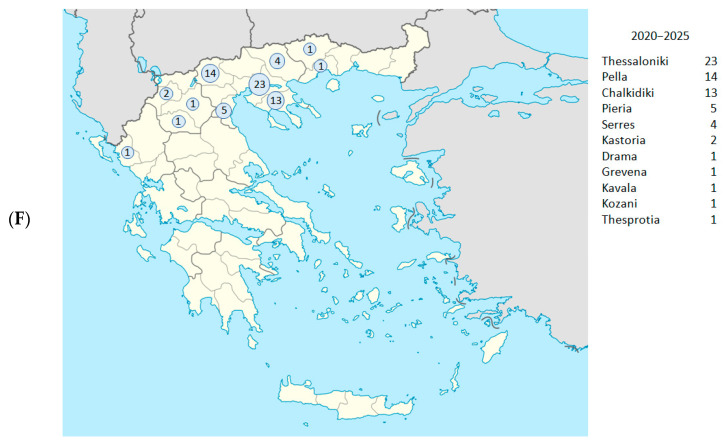
Map of Greece showing the number of samples included in the study: from 2020 (**A**); from 2021 (**B**); from 2022 (**C**); from 2023 (**D**); from 2024–2025 (**E**); and in total (**F**).

Figure 1 displays a map of Greece with the number of samples included in the study per year and in total.

**Figure 2:**
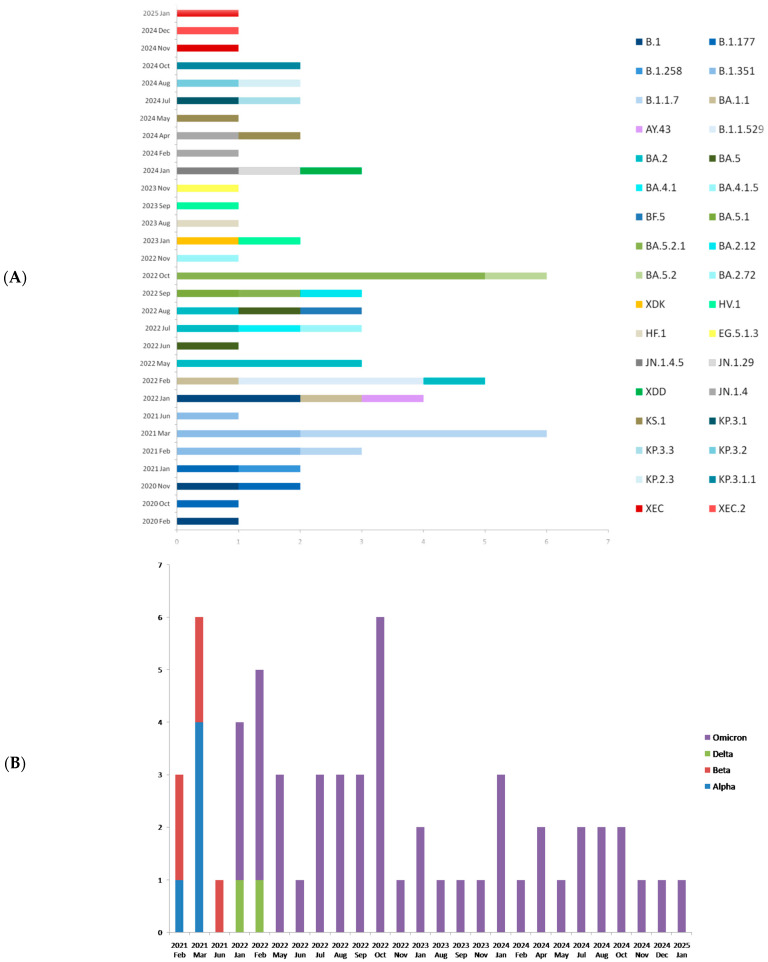

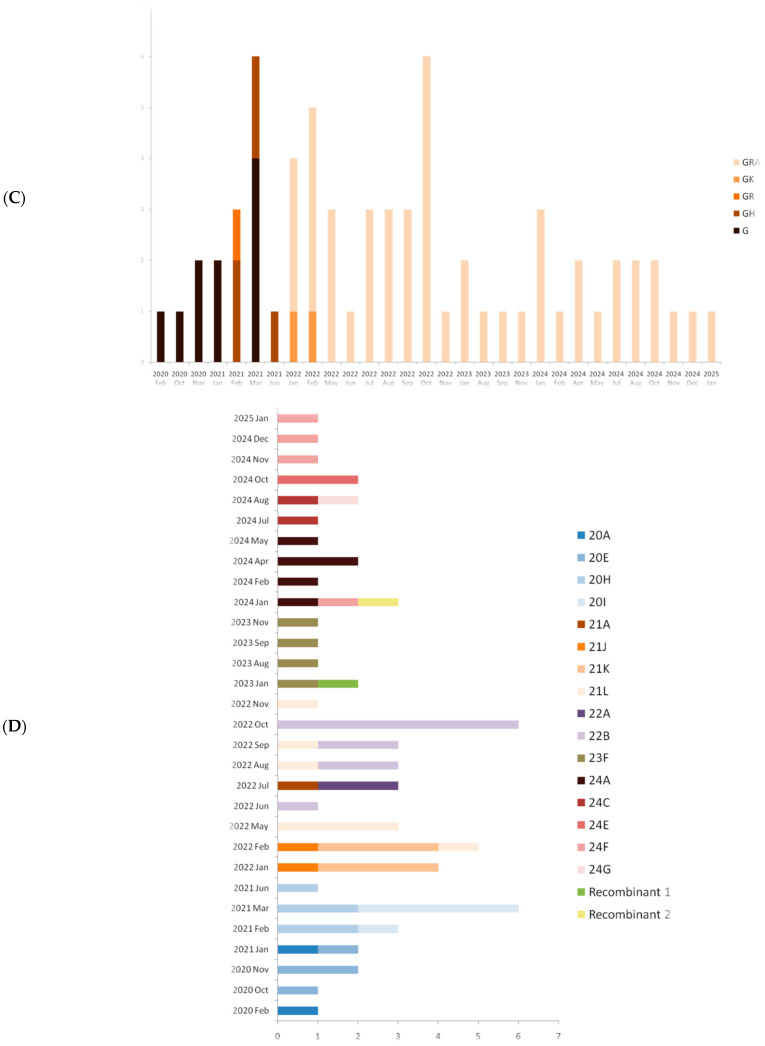
(**A**) Distribution of PANGO lineages identified throughout the study period. (**B**)Variants of concern identified in the study. (**C**) GISAID clades of the samples included in the study. (**D**) Nextstrain clades of the samples included in the study.

Thirty-four different lineages were identified according to the PANGO lineage system (Figure 2), highlighting the continuous evolution of the virus in northern Greece during the five years of the study. The VOCs according to the WHO nomenclature were Alpha and Beta between February and June 2021 (5/66 each); samples before February 2021 were not assigned as VOCs. Delta (AY.43) appeared briefly in January–February 2022 (2/66; 22.2% of genomes in that period) but was rapidly displaced by Omicron, which accounted for 77.8% (7/9) of genomes in January–February 2022 and reached 100% by May 2022. Omicron subsequently diversified into BA.1/BA.1.1 (3/66), BA.2 (6/66), BA.4/BA.5 (14/66), BF.5 (1/66), EG.5 (1/66), JN.1 (4/66), KS.1 (2/66), KP.3 (5/66), and recombinants XDK, XDD, and XEC (5/66). A year-by-year summary of lineage counts is provided in Supplementary Table S1.

## 4. Discussion

The present study provides a detailed molecular epidemiological analysis of SARS-CoV-2 circulation in various time periods in northern Greece, spanning from the first recorded COVID-19 case in February 2020 through early 2025. By employing Nanopore sequencing with the MinION Mk1C and the ARTIC protocol, we were able to track the evolution and succession of viral lineages across a five-year period in our geographic area. Our results demonstrate the dynamic nature of SARS-CoV-2, from early variants, such as Alpha and Beta, to the global dominance of Omicron and its sub-lineages, as well as the emergence of recombinant forms. These findings are consistent with the global epidemiological observations.

The first confirmed case of COVID-19 in Greece was identified in Thessaloniki on 26 February 2020 and was detected in our lab (Specimen ID 141). The patient had returned from Italy, where the COVD-19 outbreak had already initiated [12]. Soon, the first pandemic wave was initiated in Greece, and lasted until 3 May 2020 [13]. According to the present study, this first sequence belonged to the B.1 lineage, in alignment with other virus introductions in Europe from Italy that time [14]. It is classified within GISAID clade G, defined by the D614G spike mutation, which results from an A-to-G nucleotide substitution at position 23,403 in the Wuhan reference strain [15]. This mutation rapidly became dominant in the first half of 2020 due to enhanced transmissibility [16]. The detection of B.1.177 and B.1.258 lineages in late 2020 further reflects the European epidemiological pattern, as these variants were widespread across the continent during the second wave [17,18]. Accordingly, the Nextstrain 20E (EU1 cluster) that derived from 20A by an additional mutation (spike A222V) consisted of lineage B.1.177 and its sub-lineages [7], underscoring the importance of cross-border virus circulation. Interestingly, the B.1.1.318 that was notably prevalent in Greece in early 2021 [19] is absent from our dataset, probably due to our hospital-based sampling.

Our study demonstrates that by early 2021, the Alpha (B.1.1.7) and Beta (B.1.351) variants circulated in northern Greece. The Alpha variant, first identified in the United Kingdom, was associated with increased transmissibility and higher viral loads [20,21], and soon became predominant across Europe [22]. In parallel, the Beta variant, first described in South Africa, was of particular concern due to immune escape mutations (K417N, E484K, N501Y) in the spike protein [23]. The simultaneous detection of both variants locally suggests multiple introductions and heterogeneous transmission chains, as has been reported in other European countries [24].

The global spread of the Delta variant (B.1.617.2 and sub-lineages) marked a turning point in the pandemic, associated with higher transmissibility and increased risk of severe disease compared to Alpha [22,25]. In our dataset, Delta lineages such as AY.43 were identified in 2022. Moreover, our results highlight the rapid replacement of Delta by Omicron that occurred between late 2021 and early 2022 in Greece [26] (BA.1 detected in January 2022 and BA.2 in February 2022). This pattern mirrors the global trend, as Omicron rapidly achieved dominance due to its substantial immune evasion capabilities [27]. While Omicron was associated with reduced severity compared to Delta [28], its high transmissibility and the sheer number of infections led to significant morbidity and mortality, especially among unvaccinated and high-risk individuals [29].

Our study captured the progressive diversification of Omicron into BA.4.1, BA.5, and subsequent sub-lineages, including BF.5, EG.5, JN.1, KS.1, and KP.3. These findings underscore the virus’s ongoing adaptability in the face of widespread vaccination and natural immunity. Recent data suggest that Omicron sub-lineages exhibit convergent evolution at key antigenic sites, such as receptor-binding domain (RBD) mutations facilitating immune escape [30]. The detection of recombinant lineages in our dataset is also consistent with global reports of recombination as a significant evolutionary mechanism in SARS-CoV-2 [31]. Recombination can confer selective advantages by combining immune-evasive and transmissible features from different parental strains, warranting continuous genomic surveillance. In line with WHO’s current framework, these are described by their PANGO names and were classified as VOI or VUM during 2023–2025, without assignment of new Greek letters. Epidemiologically, EG.5 was designated a WHO VOI on 9 August 2023 [32], JN.1 became globally dominant and informed 2024–2025 vaccine updates [33], and KP.3/KP.3.1.1 were prominent “FLiRT” lineages during 2024, while KS.1/KS.1.1 remained less prevalent PANGO lineages [34].

The use of Oxford Nanopore technology (ONT) allowed rapid and cost-effective sequencing, even under resource-limited conditions. Nanopore sequencing is increasingly adopted for real-time outbreak monitoring due to its portability and ability to generate complete viral genomes within hours [35,36]. While Illumina sequencing remains the gold standard for accuracy, ONT has proven valuable for genomic surveillance, particularly when rapid turnaround is essential [37]. In our study, ONT enabled continuous monitoring across a five-year period, supporting its feasibility for surveillance in specialized or even decentralized laboratories. Furthermore, integration with open-source tools facilitated lineage assignment and contextualization within global phylogenies.

Some limitations of the study must be acknowledged. For a five-year period, the sample size was relatively small (*n* = 66), thus limiting the representativeness of the findings. Larger-scale sequencing efforts would better capture the full diversity of circulating lineages. Second, sampling was biased mostly towards hospitalized patients, which may not fully reflect community-level transmission dynamics. Third, given the fast pace of viral evolution, some transient lineages may have gone undetected due to limited temporal and geographical sampling. Finally, while ONT sequencing provides rapid data, it is prone to higher error rates compared with Illumina platforms [38].

Despite these limitations, the present study demonstrates the value of continuous genomic surveillance in understanding the evolutionary trajectory of SARS-CoV-2. Our findings reinforce the necessity of sustaining sequencing capacity beyond the acute pandemic phase. Genomic monitoring is particularly critical for detecting immune-escape variants, recombinants and lineages with altered clinical or epidemiological characteristics. The recent establishment of WHO’s CoViNet provides an opportunity to integrate regional surveillance efforts into a global framework, enabling rapid data sharing and coordinated response strategies.

The COVID-19 pandemic prompted the application of new sequencing technologies and bioinformatic programs which can be used for additional diseases as molecular epidemiology carries significant implications for public health policy. The dynamic replacement of lineages and the repeated introduction of variants into northern Greece illustrate that viral evolution cannot be considered independent from human mobility, population immunity, and intervention strategies. Regional outbreaks were often linked to international travel and local social dynamics, suggesting that timely genomic data, when integrated with epidemiological investigations, can provide actionable insights for containment. Strengthening the interface between genomic surveillance and public health decision-making could shorten response times to emerging threats.

In addition, the gradual transition from the pandemic emergency phase to endemic circulation of SARS-CoV-2 underscores the need to reshape surveillance frameworks. Rather than maintaining virus-specific monitoring systems, integrated platforms that simultaneously track SARS-CoV-2, influenza, respiratory syncytial virus (RSV), and other respiratory viruses provide a more sustainable and cost-effective model [39]. Currently the genomic surveillance is integrated in Greece with other respiratory viruses, such as influenza and RSV, to establish comprehensive respiratory virus surveillance networks.

The role of recombination in SARS-CoV-2 evolution warrants particular attention, as recombinants may pose unpredictable risks. In addition, the combination of genomic data with serological studies and vaccination records can provide insights into the interplay between immunity and viral evolution.

Numerous studies, especially during the COVID-19 pandemic, have shown the value of portable and rapid sequencing technologies. Especially when the results are combined with open-access databases, these can establish resilient surveillance networks capable of detecting convergent evolution, recombination, and immune-escape mutations in real time. The lessons learned from SARS-CoV-2 emphasize that preparedness requires not only technological readiness, but also sustained investment, international collaboration, and transparent data sharing. By embedding genomic surveillance within broader public health infrastructure, countries can move from reactive crisis management toward proactive prevention of future pandemics. In general, the lessons learned from SARS-CoV-2 genomic surveillance could be applied proactively to prepare for potential future pandemics caused by novel coronaviruses or other emerging pathogens.

## 5. Conclusions

This study provides a comprehensive molecular epidemiological overview of SARS-CoV-2 circulation in northern Greece over a five-year period starting from the index Greek case. By employing Nanopore sequencing with the ARTIC protocol, we were able to document the successive waves of viral variants, from early B.1 lineages to the global dominance of Omicron and its sub-lineages, as well as the emergence of recombinant forms. The evolutionary course of SARS-CoV-2 is shaped by many factors including international travel, regional connectivity, local transmission dynamics, as well as the influence of public health measures and vaccination campaigns. The simultaneous presence of multiple lineages reflects heterogeneous transmission chains and underlines the importance of continued genomic surveillance. In conclusion, sustained genomic monitoring, integrated with epidemiological data, remains essential to detect emerging variants, guide public health responses and strengthen pandemic preparedness.

## Figures and Tables

**Table 1:** Characteristics of the samples included in the study.

Date (Day/ Month/Year)	Year- Week	Sample ID	Regional Unit of Residence	Hospital	PANGO Lineage	WHO Label	GISAID Clade	NextstrainClade
29 February 2020	2020-W09	141	Thessaloniki	AHEPA	B.1	-	G	20A
29 October 2020	2020-W44	Β10880	Thessaloniki	Genimmatas	B.1.177	-	G	20E
23 November 2020	2020-W48	Γ4444	Thessaloniki	Genimmatas	B.1	-	G	20E
22 November 2020	2020-W47	Γ4452	Thessaloniki	Genimmatas	B.1.177	-	G	20E
28 January 2021	2021-W04	Δ4991	Thessaloniki	Agios Dimitrios	B.1.177	-	G	20E
28 January 2021	2021-W04	Δ5147	Kavala	Kavala	B.1.258	-	G	20A
19 February 2021	2021-W07	Δ8410	Thessaloniki	Ippokrateio	B.1.351	Beta	GH	20H
20 February 2021	2021-W07	Δ8608	Thessaloniki	Ippokrateio	B.1.351	Beta	GH	20H
27 February 2021	2021-W08	Δ9766	Thessaloniki	Ippokrateio	B.1.1.7	Alpha	GR	20I
1 March 2021	2021-W07	Pg61	Thessaloniki	Papageorgiou	B.1.1.7	Alpha	G	20I
1 March 2021	2021-W09	E109	Thessaloniki	Ippokrateio	B.1.351	Beta	GH	20H
5 March 2021	2021-W09	E926	Pieria	Katerini	B.1.351	Beta	GH	20H
12 March 2021	2021-W10	E1776	Drama	Drama	B.1.1.7	Alpha	G	20I
31 March 2021	2021-W13	E4748	Pella	Giannitsa	B.1.1.7	Alpha	G	20I
17 March 2021	2021-W11	Pg91	Thessaloniki	Papageorgiou	B.1.1.7	Alpha	G	20I
10 June 2021	2021-W23	Ip21	Thessaloniki	Ippokrateio	B.1.351	Beta	GH	20H
14 January 2022	2022-W02	O5490	Grevena	Grevena	BA.1.1	Omicron	GRA	21K
14 January 2022	2022-W02	O5475	Pieria	Katerini	BA.1	Omicron	GRA	21K
15 January 2022	2022-W02	O5476	Pieria	Katerini	BA.1	Omicron	GRA	21K
21 January 2022	2022-W03	O6666	Pieria	Katerini	AY.43	Delta	GK	21J
1 February 2022	2022-W05	Π322	Thessaloniki	AHEPA	AY.43	Delta	GK	21J
23 February 2022	2022-W08	Π2997	Thessaloniki	Papageorgiou	B.1.1.529	Omicron	GRA	21K
23 February 2022	2022-W08	Π3004	Serres	Serres	B.1.1.529	Omicron	GRA	21K
24 February 2022	2022-W08	Π3005	Kastoria	Kastoria	B.1.1.529	Omicron	GRA	21K
28 February 2022	2022-W09	Π3147	Chalkidiki	Poligiros	BA.2	Omicron	GRA	21L
18 May 2022	2022-W16	T269	Chalkidiki	Poligiros	BA.2	Omicron	GRA	21L
23 May 2022	2022-W16	Τ372	Kastoria	Kastoria	BA.2	Omicron	GRA	21L
26 May 2022	2022-W17	T374	Chalkidiki	Poligiros	BA.2	Omicron	GRA	21L
31 May 2022	2022-W17	Υ4	Pieria	Katerini	BA.5	Omicron	GRA	22B
31 May 2022	2022-W17	Υ34	Serres	Serres	BA.4.1	Omicron	GRA	22A
12 July 2022	2022-W28	Φ216	Thessaloniki	Ippokrateio	BA.4.1.5	Omicron	GRA	22A
14 July 2022	2022-W28	Φ437	Thessaloniki	Papanikolaou	BA.2	Omicron	GRA	21A
21 August 2022	2022-W33	Υ220	Chalkidiki	Poligiros	BA.5	Omicron	GRA	22B
1 August 2022	2022-W31	Χ115	Chalkidiki	Poligiros	BA.2	Omicron	GRA	21L
1 August 2022	2022-W31	Χ148	Pella	Edessa	BF.5	Omicron	GRA	22B
5 September 2022	2022-W36	Ψ68	Serres	Serres	BA.5.1	Omicron	GRA	22B
2 September 2022	2022-W35	Ψ60	Chalikidiki	Poligiros	BA.5.2.1	Omicron	GRA	22B
9 September 2022	2022-W36	Ψ157	Thessaloniki	Papanikolaou	BA.2.12	Omicron	GRA	21L
6 October 2022	2022-W40	Ω208	Thessaloniki	Papanikolaou	BA.5.2.1	Omicron	GRA	22B
6 October 2022	2022-W40	Ω210	Thessaloniki	Papanikolaou	BA.5.2.1	Omicron	GRA	22B
26 October 2022	2022-W43	Ω426	Chalkidiki	Poligiros	BA.5.2	Omicron	GRA	22B
23 October 2022	2022-W42	Ω375	Chalkidiki	Poligiros	BA.5.2.1	Omicron	GRA	22B
28 October 2022	2022-W43	Ω447	Chalkidiki	Poligiros	BA.5.2.1	Omicron	GRA	22B
26 October 2022	2022-W43	Ω410	Chalkidiki	Poligiros	BA.5.2.1	Omicron	GRA	22B
1 November 2022	2022-W44	1A-10	Chalkidiki	Poligiros	BA.2.72	Omicron	GRA	21L
12 January 2023	2023-W02	S1	Thessaloniki	Ippokrateio	XDK	Omicron	GRA	Recombinant 1
14 January 2023	2023-W02	D1	Thessaloniki	Ippokrateio	HV.1	Omicron	GRA	23F
3 August 2023	2023-W31	C4172	Kozani	Kozani	HF.1	Omicron	GRA	23F
13 September 2023	2023-W37	1Λ-10	Chalkidiki	Poligiros	HV.1	Omicron	GRA	23F
17 November 2023	2023-W46	C5066	Thessaloniki	Ippokrateio	EG.5.1.3	Omicron	GRA	23F
22 January 2024	2024-W04	C5660	Thesprotia	Filiaton	JN.1.4.5	Omicron	GRA	24F
5 January 2024	2024-W01	C5495	Pella	Edessa	JN.1.29	Omicron	GRA	24A
21 January 2024	2024-W51	C5422	Serres	Serres	XDD	Omicron	GRA	Recombinant 2
1 February 2024	2024-W05	1Λ353	Pella	Giannitsa	JN.1.4	Omicron	GRA	24A
8 April 2024	2024-W06	1Λ554	Pella	Giannitsa	JN.1.4	Omicron	GRA	24A
21 April 2024	2024-W16	1Λ593	Pella	Giannitsa	KS.1	Omicron	GRA	24A
2 May 2024	2024-W18	1Λ638	Pella	Giannitsa	KS.1	Omicron	GRA	24A
14 July 2024	2024-W28	1Λ761	Pella	Giannitsa	KP.3.1	Omicron	GRA	24C
29 July 2024	2024-W31	1Λ786	Pella	Giannitsa	KP.3.3	Omicron	GRA	24C
6 August 2024	2024-W32	C5926	Pella	Edessa	KP.3.2	Omicron	GRA	24C
6 August 2024	2024-W32	C5927	Pella	Edessa	KP.2.3	Omicron	GRA	24G
7 October 2024	2024-W41	1Λ867	Pella	Giannitsa	KP.3.1.1	Omicron	GRA	24E
5 October 2024	2024-W40	C6016	Thessaloniki	Ippokrateio	KP.3.1.1	Omicron	GRA	24E
21 November 2024	2024-W47	1Λ907	Pella	Giannitsa	XEC	Omicron	GRA	24F
27 November 2024	2024-W48	C6098	Pella	Edessa	XEC.2	Omicron	GRA	24F
7 January 2025	2025-W02	C6183	Chalkidiki	Poligiros	XEC	Omicron	GRA	24F

## Data Availability

The data presented in this study are available in the article.

## Supplementary Materials

The following supporting information can be downloaded at: https://www.mdpi.com/article/10.3390/epidemiologia6040078/s1, Table S1: Year-by-year summary of PANGO lineage counts.
